# Periostin in diabetic kidney disease: mechanistic insights, biomarkers, and therapeutic perspectives

**DOI:** 10.3389/fendo.2026.1979232

**Published:** 2026-10-09

**Authors:** Xuhao Li, Yamiao Wu, Yexuan Shi, Guoliang Xiong, Lingfei Lu, Jiandong Lu

**Affiliations:** 1 The Fourth Clinical Medical College of Guangzhou University of Chinese Medicine, Shenzhen, Guangdong, China; 2 Department of Nephrology, Shenzhen Traditional Chinese Medicine Hospital, Shenzhen, Guangdong, China

**Keywords:** biomarker, diabetic kidney disease, periostin, renal fibrosis, therapeutic perspectives

## Abstract

Diabetic kidney disease (DKD) is among the most common and severe microvascular complications of diabetes and represents a leading cause of end-stage renal disease (ESRD) worldwide. Currently, although strict glycemic and blood pressure control, together with the use of renin–angiotensin system inhibitors, sodium-glucose cotransporter 2 (SGLT2) inhibitors, glucagon-like peptide-1 (GLP-1) receptor agonists, and non-steroidal selective mineralocorticoid receptor antagonists, can moderately delay DKD progression, the risks of progression to ESRD and cardiovascular events remain high, highlighting the urgent need to identify novel therapeutic targets. Preclinical and observational studies suggest that periostin (POSTN) is associated with DKD progression and may contribute to renal inflammation and fibrosis. POSTN expression is minimal in healthy kidneys but is markedly upregulated under conditions of renal injury and fibrosis. Cross-sectional clinical studies report that serum, urinary, and renal tissue POSTN levels are elevated in patients with DKD and correlate with proteinuria, the severity of renal fibrosis, and a decline in estimated glomerular filtration rate (eGFR); these associations have not been confirmed prospectively. Preclinical studies indicate that POSTN promotes inflammatory cell infiltration, myofibroblast activation, and extracellular matrix accumulation, and it has therefore been proposed to contribute to the inflammatory and fibrotic progression of DKD. In preclinical DKD models, POSTN inhibition attenuates renal injury and fibrosis. No POSTN-targeted therapy has entered human clinical trials, so POSTN remains a promising preclinical candidate target. This review summarizes the mechanistic basis and recent advances in POSTN research in DKD, and further explores its translational potential as a biomarker and therapeutic target.

## 1 Introduction

Diabetic kidney disease (DKD) is one of the most serious microvascular complications of Diabetes Mellitus (DM) and represents the leading cause of End-stage Renal Disease (ESRD) worldwide, accounting for approximately 40–50% of all patients receiving renal replacement therapy (1). In recent years, with the rising prevalence of DM, the disease burden of DKD has risen substantially. Approximately 20–50% of patients with DM eventually develop DKD, and the prevalence of DKD is projected to double over the next two decades (2–5). The pathogenesis of DKD is highly complex and involves the interplay of multiple pathological pathways, including hyperglycemia-induced metabolic disturbances, hemodynamic alterations, chronic inflammation, and oxidative stress. These factors collectively promote glomerulosclerosis, tubular atrophy, and interstitial fibrosis, ultimately culminating in progressive deterioration of renal function (6–8). Although renin-angiotensin-aldosterone system (RAAS) blockers, sodium–glucose cotransporter 2 (SGLT2) inhibitors, glucagon-like peptide-1 receptor agonists (GLP-1 RAs), and non-steroidal selective mineralocorticoid receptor antagonists can reduce the risk of renal disease progression and cardiovascular mortality by 25–40%, yet approximately 30.4% of patients still progress to ESRD (9). Furthermore, the residual risk of cardiovascular events remains unacceptably high, underscoring the urgent need for novel therapeutic strategies (9, 10). DKD also carries a substantial burden of complications beyond renal function loss; anemia is common as kidney function declines and requires specific management (11). Therefore, the identification of novel biomarkers and therapeutic targets is critically needed to improve clinical outcomes and long-term prognosis in patients with DKD.

POSTN is a multifunctional matricellular protein that is markedly upregulated under conditions of tissue injury, inflammation, and fibrosis (12, 13). In several observational human studies and preclinical DKD models, POSTN expression has been associated with renal fibrosis and renal functional decline; these associations require prospective validation (14–16). General renal fibrosis models and non-renal matrix studies provide biological context for these associations and do not establish DKD specificity. POSTN may contribute to the pathological progression of DKD through multiple mechanisms, including extracellular matrix (ECM) remodeling, activation of pro-fibrotic signaling cascades, and epithelial-mesenchymal transition (EMT)-like changes, mechanisms that are also implicated in other kidney diseases and in non-renal fibrotic models (17, 18). The extent to which classical EMT directly generates interstitial myofibroblasts in human DKD remains debated. Clinical studies report elevated serum and urinary POSTN levels in patients with DKD, with positive correlations to the urinary albumin excretion rate (UAER), the rate of eGFR decline, and the extent of renal histopathological injury. Dynamic changes in urinary POSTN levels may precede detectable abnormalities in conventional renal function parameters, which makes urinary POSTN a candidate early biomarker for the diagnosis and prognostic evaluation of DKD (19–21). Whether tubular epithelial cells give rise to interstitial myofibroblasts through EMT-like changes in patients with DKD has not been established. Findings in IgA nephropathy, a non-DKD kidney disease, support a broader link between urinary POSTN and renal injury; they do not establish DKD specificity. This review aims to systematically summarize the most recent advances in research on POSTN in DKD, with a particular focus on its mechanistic role in pathogenesis, potential diagnostic utility, and therapeutic implications, with the aim of providing novel insights to inform the clinical management and treatment of DKD.

Although POSTN is broadly induced by tissue injury, inflammation, and fibrosis, increased POSTN is not specific to diabetes or DKD. This review distinguishes DKD-specific evidence, derived from human DKD samples, diabetic animal models, or high-glucose-treated renal cells, from evidence obtained in other kidney diseases, general renal fibrosis models, or non-renal systems. The latter serves as biological context and is explicitly identified as such. Findings from streptozotocin-induced and genetically determined animal models reproduce key metabolic and structural features of diabetes but do not capture the heterogeneity of human type 1 or type 2 diabetes.

## 2 Review approach

This article is a narrative review. Relevant literature was identified through targeted searches of PubMed, Web of Science, and Embase from database inception through September 2026. The main search concepts combined periostin or POSTN with diabetic kidney disease, diabetic nephropathy, renal fibrosis, inflammation, biomarker, urine, serum, and therapy. Recent clinical guidelines, systematic reviews, meta-analyses, and human clinical studies were prioritized when biomarker performance and clinical management were assessed. Earlier landmark studies were retained when they provided foundational evidence on the physiological and molecular functions of POSTN. Animal and cellular studies were used to interpret mechanistic pathways when human evidence was limited, and the reference lists of retrieved reviews were screened for additional primary studies.

Literature identified through these searches was considered for inclusion when it addressed one of the four domains of this review: POSTN biology, the pathogenesis of DKD, biomarker performance, and therapeutic targeting. Evidence was integrated by domain, with particular attention to inconsistent findings and unresolved questions. This approach provides a mechanistically oriented narrative synthesis and was not designed as a systematic review or meta-analysis. Representative studies were selected purposively from the identified human and preclinical studies, with priority given to those reporting direct and interpretable evidence on renal outcomes, biomarker performance, or POSTN-targeted intervention. The studies summarized in **Tables 1**, **2** illustrate the evidence base and do not constitute a comprehensive list of all identified studies.

**Table 1:** Human biomarker studies of POSTN in DKD.

Study	Design and sample size	Population and DKD stage/definition	Specimen and assay method	Main finding	Diagnostic performance	Key limitation
Varatharajan et al., 2024 (105)	Hospital-based cross-sectional; 160 participants (40 healthy controls, 120 with T2DM)	T2DM; normoalbuminuria (n=40), microalbuminuria (n=40) and macroalbuminuria (n=40) defined by uACR; CKD stages 1–4 by eGFR	Urine; ELISA	Urinary POSTN rose stepwise across albuminuria stages, was positively correlated with ACR and negatively with eGFR, and was lower in CKD stage 1 than in stages 3-4	ROC cutoff 37.5 ng/mL; sensitivity 80%, comparable to ACR (80%) and higher than NGAL (75.8%) and KIM-1 (63.3%); specificity, AUC and predictive values not reported	Cross-sectional, single center; no external validation; specificity and AUC not reported
Ghafar et al., 2020 (106)	Cross-sectional; 137 patients with T2DM and 41 healthy controls	T2DM; normoalbuminuria, incipient nephropathy (microalbuminuria) and overt nephropathy (macroalbuminuria) defined by uACR	Urine; ELISA	Urinary POSTN was higher in patients with micro- and macroalbuminuria than in normoalbuminuric T2DM, and was correlated with the degree of albuminuria	AUC 0.954 for incipient and 0.997 for overt nephropathy, higher than urinary cyclophilin A (0.914 and 0.937); diagnostic cutoff not reported	Cross-sectional, single center; no prospective validation
Taha et al., 2022 (107)	Case-control; 102 patients with T2DM and 80 controls (46 controlled, 56 uncontrolled)	T2DM grouped as controlled (HbA1c <6.5%) and uncontrolled (HbA1c >6.5%); renal dysfunction assessed by ACR and eGFR	Urine and serum; assay not specified in the abstract	Urinary POSTN was higher in patients with T2DM than in controls and higher in uncontrolled than in controlled diabetes; it was positively correlated with ACR and negatively with eGFR, and remained an independent marker in multivariate analysis	Not reported	Case-control, single center; no longitudinal follow-up; performance metrics not reported
El-Dawla et al., 2019 (108)	Case-control; 71 patients with diabetic nephropathy and 19 healthy controls	Diabetic nephropathy; normoalbuminuria (n=19, UACR <30 mg/g), microalbuminuria (n=37, 30–300 mg/g) and macroalbuminuria (n=15, >300 mg/g)	Serum; ELISA	POSTN increased and E-cadherin decreased with albuminuria stage, and POSTN was positively correlated with UACR	AUC 0.833 (95% CI 0.709-0.919) for established microalbuminuria (E-cadherin AUC 0.998); cutoff not reported	single-center sample; no independent validation cohort
Patel et al., 2019 (109)	Cross-sectional, tertiary center; 360 patients with T2DM and 50 healthy controls	T2DM; normoalbuminuria (n=95), microalbuminuria (n=130) and macroalbuminuria (n=135)	Urine; ELISA	Urinary POSTN was elevated in all diabetic groups compared with controls and correlated with age, albuminuria and eGFR decline	cutoffs 2.11, 2.74 and 7.81 ng/mL for normo-, micro- and macroalbuminuria, with sensitivity/specificity of 88%/100%, 98.1%/66.7% and 100%/69.9%	Cross-sectional, single center; restricted to T2DM; no external validation
Satirapoj et al., 2015 (19)	Cross-sectional with kidney-tissue immunohistochemistry; 328 patients with T2DM and 30 healthy volunteers	T2DM; normoalbuminuria (n=114), microalbuminuria (n=100) and macroalbuminuria (n=114); kidney tissue from overt diabetic nephropathy	Urine; ELISA	POSTN expression increased in glomeruli and tubular epithelium, and urinary POSTN was elevated in all albuminuria groups compared with controls and correlated with age, albuminuria and GFR decline	AUC 0.78 for normoalbuminuria, 0.99 for microalbuminuria and 0.95 for macroalbuminuria; cutoff not reported	Cross-sectional; no independent validation cohort
Satirapoj et al., 2011 (112)	Clinical study with experimental models (5/6 nephrectomy, streptozotocin-diabetic and UUO models, tubular cells); human sample size not reported	Patients with CKD of mixed etiology and experimental kidney injury; DKD not separately defined	Urine; ELISA	Urinary POSTN excretion increased progressively after injury and was detectable in patients with CKD; POSTN induced a coordinated mesenchymal phenotype in tubular cells	cutoff 32.66 pg/mg creatinine; sensitivity 92.3% and specificity 95.0% for distinguishing CKD from healthy controls	Mixed etiology, not DKD-specific; human sample and CKD stages not reported
Ibrahim et al., 2025 (113)	Case-control; 72 patients with T2DM and 18 healthy controls	T2DM subdivided by GFR category (G1-G4); renal fibrosis assessed non-invasively	Serum; ELISA	Serum POSTN increased significantly with DKD progression and contributed to non-invasive prediction of renal fibrosis severity	cutoff 39.92 ng/mL; sensitivity 97.2% and specificity 100% for early detection of DKD (renal resistivity index cutoff 0.5, sensitivity 83.3%, specificity 100%; point shear-wave elastography (PSWE) 1.52 kPa)	single center; imaging surrogates rather than biopsy; no longitudinal validation
Al-temimi et al., 2024 (114)	Cross-sectional; 120 Iraqi men (60 with T2DM, 60 with diabetic nephropathy on hemodialysis)	T2DM without nephropathy versus diabetic nephropathy requiring hemodialysis	Serum; ELISA	Serum POSTN was significantly higher in patients with diabetic nephropathy on hemodialysis than in those with T2DM alone	cutoff 238.17 ng/mL; sensitivity 92% and specificity 90%	single center; male-only sample; restricted to advanced (dialysis) disease
Kurnik et al., 2023 (115) (non-DKD context)	Retrospective cohort (conference abstract); 105 kidney transplant recipients and 32 healthy volunteers	Kidney transplant recipients at least 2 years post-transplant; no DKD	Serum; commercial immunoassays	Median serum POSTN was lower in transplant recipients than in healthy volunteers (959 vs 1176 ng/mL), consistent with immunosuppression or impaired clearance	Not reported	Non-DKD population; does not reflect diabetic kidney injury
Hwang et al., 2016 (21) (non-DKD context)	Retrospective cohort from a glomerulonephritis cohort (2009-2014); 314 of 399 recruited patients analyzed	Biopsy-proven IgA nephropathy; no DKD	Urine and serum; ELISA	Urinary POSTN/creatinine correlated with histological fibrosis and tubular atrophy and independently predicted ESRD	Not reported	Non-DKD disease; findings cannot be extrapolated to DKD
Tan et al., 2018 (110) (non-DKD context)	Two-center cross-sectional observational study; 188 Chinese adults (120 non-asthma, 68 asthma)	Adults of Chinese descent; reference-value study, no DKD	Serum; immunoassay	Sex-specific reference ranges were established: 61.1 ng/mL in women and 53.2 ng/mL in men	Not reported	Non-DKD population; observational design
Walsh et al., 2017 (111) (non-DKD context)	Cross-sectional observational study; 166 healthy men and women	Three skeletal stages (16-18, 30–32 and over 70 years); bone-focused cohort, no DKD	Serum; ELISA	Serum POSTN was higher at 16–18 than at 30–32 years and was inversely correlated with tibial cortical thickness and density	Not reported	Non-DKD, bone-focused cohort

**Table 2:** Preclinical intervention studies targeting POSTN.

Model	Intervention	Main renal outcome	Key limitation	Reference
Diabetic mice	POSTN-binding DNA aptamer	Reduced blood urea nitrogen; attenuated tubulointerstitial fibrosis and expression of fibronectin and type I collagen	Animal model only; short follow-up; no dose-response data	(125)
Mice with unilateral ureteral obstruction (UUO)	Anti-POSTN neutralizing antibody	Attenuated renal fibrosis and reduced fibrosis- and inflammation-related gene expression	Non-diabetic kidney injury model; short follow-up; no human validation	(126)
Diabetic mice	Genetic knockout	Reduced urinary albumin excretion, glomerulosclerosis, interstitial fibrosis and fibrosis markers; improved glucose regulation, islet integrity and insulin expression	Systemic knockout; species differences; no human validation	(97)
Diabetic renal injury models	siRNA-mediated POSTN knockdown	Alleviated pathological changes and renal fibrosis	Animal model; delivery and durability not established	(17)
High-glucose-stimulated human renal tubular epithelial cells	p38/MAPK inhibitor	Reduced POSTN and α-SMA, restored E-cadherin, and reduced ECM synthesis and secretion	*In vitro* only; *in vivo* causality unproven	(100)
High-glucose-stimulated human renal tubular epithelial cells	POSTN knockdown or neutralizing antibody	Partially reversed EMT marker changes	*In vitro* only; human relevance unproven	(98)
Cystic kidney disease model	MMP-9-mediated POSTN cleavage	POSTN cleavage was protective against kidney cystic disease	Non-DKD model; opposite direction of effect, cannot be extrapolated to DKD	(127)

Each summarized study was appraised qualitatively. Design strength was determined from the feature that most increased the reliability or clinical interpretability of the evidence: prospective follow-up, adequate sample size, an appropriate comparison group, multivariate adjustment, direct histological or physiological measurement, or clinically relevant renal outcomes. The main limitation was defined as the feature most likely to restrict causal inference, precision, or generalizability: cross-sectional or case-control design, single-center sampling, small sample size, short follow-up, absence of an independent validation cohort, lack of adjustment for the urine albumin-to-creatinine ratio (ACR) or eGFR, or restriction to a specific population. Interpretation considered the design strength and the main limitation together, and each conclusion was limited to the level of inference the study design supports. Observational studies support associations; preclinical intervention studies demonstrate biological effects in models and do not establish clinical efficacy. No numerical score or formal ranking was assigned.

Throughout the manuscript, evidence is differentiated according to its source and the level of inference supported by the study design. DKD-specific evidence, derived from human DKD samples, diabetic animal models, or high-glucose-treated renal cells, is distinguished from evidence obtained in other kidney diseases, general renal fibrosis models, and non-renal systems; the latter is presented as biological context. Observational findings are reported as associations unless the design supports a stronger inference. Animal and cellular findings are used to support biological plausibility, and mechanisms without direct human validation are described as proposed or hypothesis-generating.

## 3 Overview of POSTN and diabetic kidney disease

### 3.1 Biological characteristics and functions of POSTN

POSTN is a secreted matricellular protein belonging to the fasciclin family of proteins. POSTN was first identified in osteoblast cell lines in 1993 (22). The POSTN gene, located on human chromosome 13, encodes a protein with a molecular weight of approximately 90 kDa, consisting of 836 amino acid residues (23). The protein structure of POSTN comprises several distinct functional domains: the N-terminus carries a signal peptide sequence that directs its secretion into the ECM, immediately followed by an EMI domain; the central region consists of four fasciclin-I repeat domains responsible for mediating interactions with integrin receptors and other ECM components; and the C-terminus is rich in cysteine residues, enabling the generation of multiple splice isoforms that participate in disulfide bond formation and the maintenance of protein stability (24, 25). Unlike classical ECM structural molecules such as collagen and fibronectin, POSTN does not directly serve as a structural scaffold for the ECM but acts as a “molecular bridge” to regulate communication between cells and the ECM, thereby playing a pivotal regulatory role in tissue development, injury repair, and pathological remodeling (26). POSTN expression exhibits marked spatiotemporal specificity and is subject to pathological induction. Under physiological conditions, POSTN expression is markedly elevated during embryonic development, where it participates in the morphogenesis and differentiation of organs such as cardiac valves, bones, and periodontal tissues, playing a pivotal role in cardiac mesenchymal development and bone formation (27–29); in healthy adult tissues, however, POSTN protein levels are generally low, with expression limited to collagen-rich connective tissues such as the periosteum, periodontal ligaments, and cardiac valves, in order to maintain tissue homeostasis (30). By contrast, under pathological stimuli such as tissue injury, inflammation, or fibrosis, POSTN expression can be re-induced and markedly upregulated, with predominant localization in activated myofibroblasts, injured myocardium, inflamed airways, and tumor-associated stroma (31, 32).

POSTN plays a pivotal role in the pathogenesis and progression of various diseases (**Figure 1**). In the cardiovascular system, POSTN is re-expressed in the infarct border zone and fibrotic regions following myocardial infarction. Activated cardiac fibroblasts and myofibroblasts secrete substantial amounts of POSTN, thereby promoting scar formation, the transdifferentiation of fibroblasts into myofibroblasts, and the maturation of the ECM (33, 34). POSTN activates focal adhesion kinase (FAK), phosphoinositide 3-kinase/protein kinase B (PI3K/Akt), and pro-fibrotic gene expression programs through integrin-mediated signaling pathways, while synergizing with the transforming growth factor-β (TGF-β) signaling pathway to further amplify ECM deposition and fibroblast activation (35, 36). Moderate POSTN expression is essential for maintaining scar integrity, but its overexpression leads to adverse cardiac remodeling and contributes to the progression of heart failure (37). In diabetic cardiomyopathy, POSTN is markedly upregulated through the TGF-β/Smad pathway, promoting myocardial fibrosis and cardiac dysfunction; conversely, POSTN knockout mice demonstrate a protective phenotype against DM-induced myocardial injury (38). In the respiratory system, POSTN acts as a downstream effector of type 2 helper T-cell cytokines and is implicated in the development of asthma phenotypes, airway remodeling, and fibrotic lung diseases. Airway epithelial cells produce POSTN upon stimulation with interleukin (IL)-4/IL-13; serum POSTN levels correlate with the clinical features of type 2 asthma and may serve as a predictive biomarker for responsiveness to anti-IL-13 or anti-immunoglobulin E (IgE) therapy (39, 40). In idiopathic pulmonary fibrosis, POSTN promotes fibroblast activation, ECM deposition, and structural airway remodeling, thereby driving the fibrotic remodeling process (41). In tumor biology, POSTN is frequently overexpressed in the tumor stroma and cancer-associated fibroblasts (CAFs), where it facilitates EMT, promotes cell survival, and enhances tumor invasion and metastasis through integrin-mediated PI3K/Akt, FAK, extracellular signal-regulated kinase (ERK), and nuclear factor κB (NF-κB) signaling pathways (42–44). POSTN also shapes a supportive tumor microenvironment by promoting angiogenesis, recruiting M2 macrophages, and activating CAFs (45). In esophageal adenocarcinoma, for instance, POSTN secreted by CAFs binds to integrins αvβ3/αvβ5 on the surface of tumor cells, activating the PI3K/Akt pathway and thereby promoting invasion; furthermore, stromal POSTN protein levels are positively correlated with poor patient prognosis (46). In ovarian cancer, POSTN promotes tumor growth and metastasis through the integrin–ERK–NF-κB signaling axis, while recruiting M2 macrophages and activating CAFs; its elevated expression is significantly associated with advanced tumor stage and poor prognosis (47). Multiple studies have confirmed that in various malignant tumors, including breast, gastric, colorectal, and pancreatic cancers, POSTN frequently promotes EMT, sustains cancer stem cell properties, induces angiogenesis, and drives metastasis through signaling pathways such as PI3K/Akt, FAK, and Wnt signaling pathways (48, 49). In the skeletal system, POSTN is essential for bone formation, periosteal response, and fracture repair. POSTN is highly expressed in the periosteum and cortical bone, playing a pivotal role in bone development and the maintenance of bone homeostasis. POSTN-expressing bone marrow-derived macrophage populations can drive osteogenesis and angiogenesis, promoting M2 macrophage polarization and angiogenesis–osteogenesis coupling in bone repair models, thereby improving cortical bone repair (50). During skin wound healing, POSTN serves as a pivotal stromal mediator in both the proliferative and remodeling phases, regulating fibroblast behavior, collagen assembly, and re-epithelialization (51).

**Figure 1:**
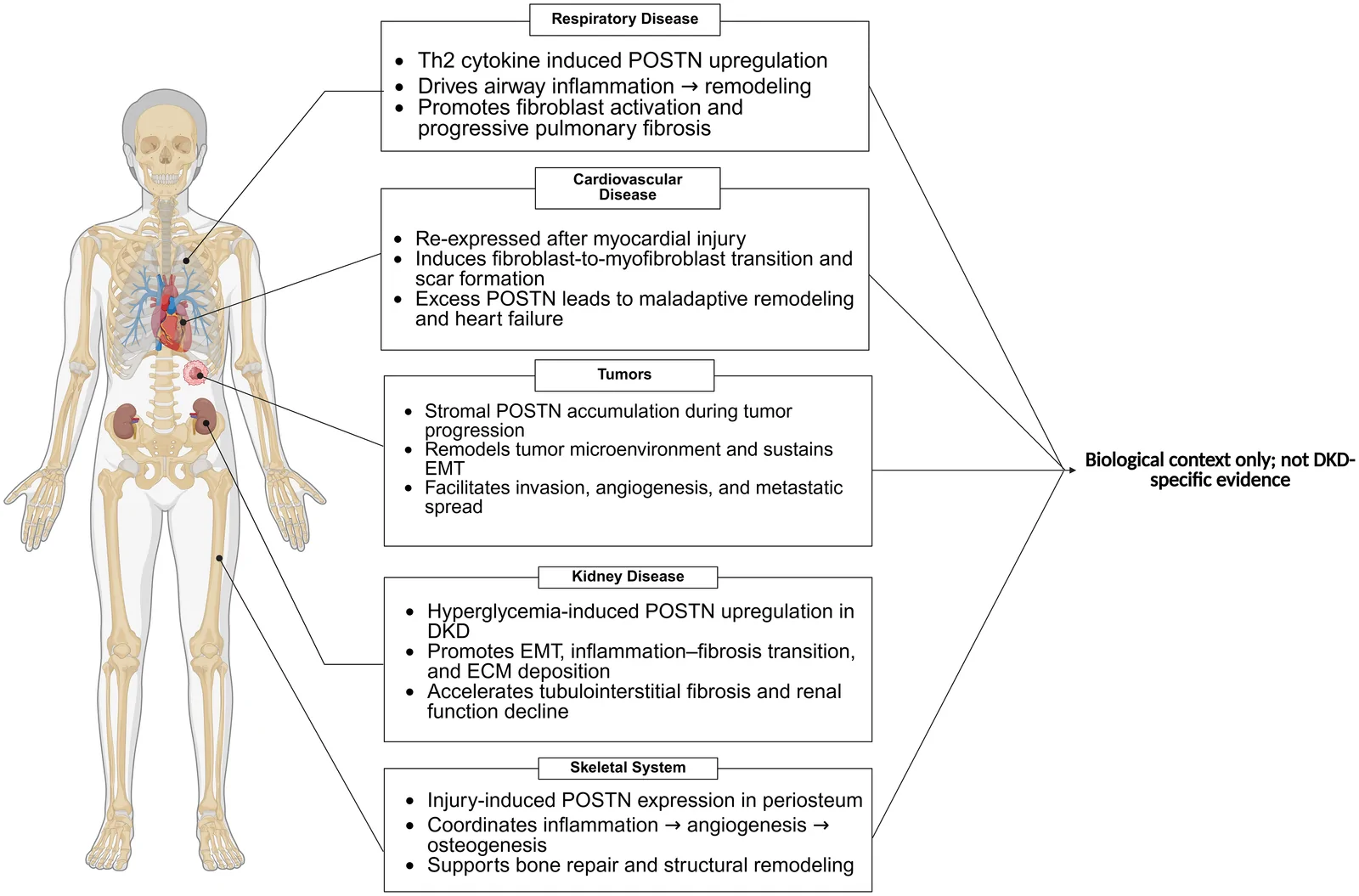
Biological characteristics and pathological functions of POSTN.

POSTN is a matricellular protein predominantly secreted by activated fibroblasts and epithelial cells under pathological stress. Through interactions with integrins and extracellular matrix components, POSTN regulates inflammation, tissue remodeling, epithelial–mesenchymal transition, and fibrosis across multiple diseases.

### 3.2 Pathological mechanisms of diabetic kidney disease

The pathogenesis of DKD arises from the combined effects of metabolic abnormalities and hemodynamic changes, which act synergistically to activate multiple downstream signaling pathways and induce pathological remodeling of renal cells and the surrounding tissue architecture. Metabolic abnormalities primarily manifest as chronic hyperglycemia and its associated glucolipotoxicity, whereas hemodynamic changes are characterized by glomerular hyperfiltration and aberrant activation of the RAAS. These two major drivers, through multiple converging pathological mechanisms, including inflammatory responses, oxidative stress, and mitochondrial dysfunction, ultimately culminate in cellular injury, programmed cell death, and fibrosis of renal parenchymal cells, resulting in irreversible deterioration of renal function (**Figure 2**).

**Figure 2:**
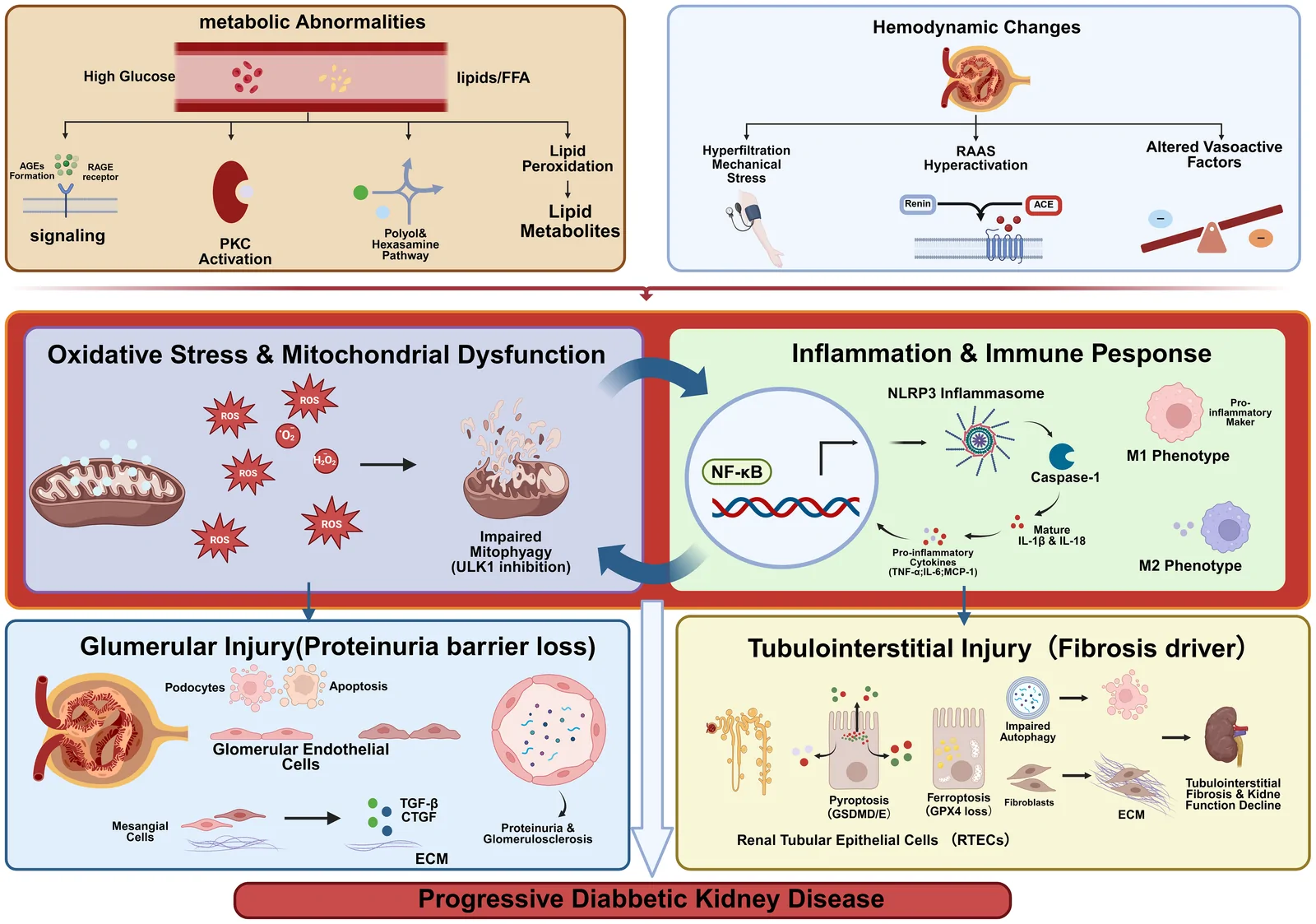
Pathogenic mechanisms underlying DKD progression.

#### 3.2.1 Pathways of cellular damage induced by metabolic abnormalities

Chronic hyperglycemia disrupts normal renal cellular metabolism through multiple interconnected metabolic pathways, thereby impairing renal tissue structure and function (52). The accumulation of advanced glycation end products (AGEs) represents one of the central pathogenic mechanisms underlying the pathogenesis of DKD (53). Upon binding to the receptor for advanced glycation end products (RAGE), AGEs activate downstream NF-κB and p38 mitogen-activated protein kinase (p38/MAPK) signaling pathways, thereby promoting the expression of inflammatory mediators and aberrant ECM deposition (54). The accumulation of AGEs not only alters the composition and structure of the glomerular basement membrane and mesangial ECM but also upregulates TGF-β expression, thereby mediating excessive matrix deposition and glomerular hypertrophy (55). Concurrently, protein kinase C (PKC) is markedly activated under hyperglycemic conditions, thereby increasing vascular permeability, inducing hemodynamic disturbances, and upregulating the expression of pro-fibrotic factors (56). Furthermore, aberrant activation of the polyol pathway results in intracellular sorbitol accumulation, thereby causing elevated intracellular osmotic pressure and redox imbalance; meanwhile, augmented flux through the hexosamine pathway modulates gene expression and cellular signal transduction through O-glycosylation of proteins (57). Collectively, dysregulation of these metabolic pathways constitutes the molecular basis of glycotoxicity, thereby providing the initiating signals for downstream inflammatory responses and oxidative stress. In addition to hyperglycemia, lipotoxicity arising from lipid metabolism dysregulation represents an additional key driver of DKD pathogenesis. Lipids aberrantly deposited within renal tubular epithelial cells can induce lipid peroxidation, thereby generating an abundance of reactive lipid metabolites. These metabolites further activate pro-inflammatory signaling pathways, thereby exacerbating tubular damage and interstitial fibrosis (58, 59). In summary, multiple pathways, including high-glucose-induced AGEs, PKC, polyols, and hexosamines, collectively constitute an interconnected molecular network of glycotoxicity. Combined with the synergistic effects of lipotoxicity, these pathways collectively initiate a pathological cascade of renal cellular injury.

#### 3.2.2 Hemodynamic changes

Hemodynamic abnormalities represent another key driver of DKD pathogenesis. In the early stages of DM, glomerular hyperfiltration constitutes a hallmark hemodynamic feature. This hemodynamic state imposes sustained mechanical stress on podocytes and glomerular endothelial cells, thereby leading to structural and functional impairment of the glomerular filtration barrier (60). Concurrently, aberrant activation of the RAAS leads to markedly elevated angiotensin II (Ang II) levels. Ang II not only directly constricts the afferent arterioles, increasing intraglomerular pressure, but also promotes aberrant ECM deposition and renal interstitial fibrosis through the activation of pro-fibrotic mediators, including TGF-β and connective tissue growth factor (CTGF) (61). Furthermore, the vasoactive imbalance between vasoconstrictive and vasodilatory mediators induced by aberrant insulin signaling and RAAS activation further exacerbates mechanical stress and structural impairment of the glomerular filtration barrier, ultimately manifesting as the onset and progressive exacerbation of proteinuria. Collectively, the mechanical stress and vasoactive imbalance arising from glomerular hyperfiltration and aberrant RAAS activation represent the principal hemodynamic drivers of glomerular filtration barrier impairment and the progressive development of proteinuria (62).

#### 3.2.3 Inflammatory response

The inflammatory response constitutes a central pathological component of DKD progression, responsible for translating upstream metabolic and hemodynamic signals into tissue damage and fibrosis. In DKD, both the innate and adaptive immune systems are activated, and diverse immune cell populations and their secreted inflammatory mediators contribute to the amplification and perpetuation of the pathological process. The inflammatory response involves the activation of multiple pro-inflammatory signaling pathways, including the NF-κB, Janus kinase/signal transduction and transcription activator (JAK/STAT), and NOD-like receptor family with heat shock protein-like domain 3 (NLRP3) inflammasome axes (63–65). The extent of activation of these pathways is significantly correlated with proteinuria severity and the degree of renal dysfunction across both experimental animal models and clinical patient cohorts (63, 64). As a key transcription factor, NF-κB is activated by diverse upstream stimuli, including AGEs, Ang II, and ROS, thereby driving the transcriptional upregulation of pro-inflammatory cytokines, including IL-1β, IL-6, tumor necrosis factor-α (TNF-α), and monocyte chemotactic protein-1 (MCP-1) (66, 67). The assembly and activation of the NLRP3 inflammasome lead to Caspase-1 activation, thereby promoting the proteolytic maturation and secretion of IL-1β and IL-18. These cytokines not only amplify the inflammatory response but can also directly injure renal parenchymal cells (68). The release of inflammatory cytokines and chemokines recruits substantial numbers of macrophages to infiltrate the renal interstitium. In the DKD microenvironment, macrophage polarization is skewed toward a pro-inflammatory phenotype, with a predominance of M1 macrophages and a concomitant reduction in the proportion of M2 macrophages, which exert anti-inflammatory and tissue-reparative functions (69, 70). Concurrently, the efferocytic and phagocytic clearance capacity of macrophages is impaired, preventing the effective removal of apoptotic cells and cellular debris. This impairs the timely resolution of the inflammatory response, resulting in its persistence and transition into a chronic inflammatory state, which further drives the initiation and progression of renal fibrosis. Therefore, as a critical mechanistic link in the conversion of metabolic and hemodynamic abnormalities into tissue injury, the inflammatory response drives the chronic progression of DKD through immune cell infiltration and the sustained activation of pro-inflammatory signaling pathways (71).

#### 3.2.4 Oxidative stress and mitochondrial dysfunction

Oxidative stress and mitochondrial dysfunction represent key pathological mediators linking metabolic dysregulation to cellular injury; together with inflammatory responses, they collectively constitute a mutually reinforcing pathological cycle that drives the progression of DKD. A high-glucose environment markedly augments ROS production through multiple pathways (72). Mitochondrial electron transport chain hyperactivation constitutes the primary source of ROS generation. Under hyperglycemic conditions, enhanced glucose oxidation results in augmented electron leakage in the electron transport chain and consequent elevation of superoxide anion generation (73). Additionally, pro-oxidant enzyme systems, including nicotinamide adenine dinucleotide phosphate (NADPH) oxidase, are activated by inflammatory signals, further amplifying ROS generation (74). Excessive ROS accumulation triggers widespread macromolecular oxidative damage, encompassing DNA oxidation, protein carbonylation, and lipid peroxidation. These oxidative insults disrupt normal cellular structure and function, while concurrently activating multiple stress-responsive signaling pathways, including NF-κB, JAK/STAT, and PI3K/Akt (75). Persistent oxidative stress can disrupt mitochondrial membrane potential, impair mitochondrial DNA integrity and respiratory chain complex function, ultimately resulting in mitochondrial dysfunction (76). Dysfunctional mitochondria not only produce less ATP but also generate more ROS, thereby establishing a self-amplifying positive feedback loop between ROS generation and mitochondrial dysfunction (77). Under physiological conditions, damaged mitochondria are selectively recognized and cleared through mitochondria-specific autophagy (mitophagy), thereby maintaining mitochondrial homeostasis and quality control. However, in DKD, the mitophagy pathway mediated by UNC-51-like autophagy-activating kinase 1 (ULK1) is inhibited, resulting in the aberrant accumulation of dysfunctional mitochondria within renal cells, which further amplifies ROS generation and perpetuates inflammatory responses (78). Experimental animal studies have demonstrated that restoring ULK1-mediated mitophagy restores mitochondrial function and attenuates renal tissue injury, suggesting that dysregulation of mitophagy plays a pivotal role in the pathogenesis of DKD (79). In summary, oxidative stress and mitochondrial dysfunction constitute a mutually reinforcing pathological cycle, while impaired mitophagy further exacerbates this pathological process, collectively driving the pathological progression of DKD.

#### 3.2.5 Programmed cell death

Driven by the aforementioned multifaceted pathological mechanisms, renal cells may undergo diverse forms of programmed cell death, including autophagy, apoptosis, pyroptosis, and ferroptosis. These death pathways exert context-dependent dual effects in DKD: mediating either cytoprotective adaptations or tissue-injurious outcomes, with the specific consequence depending on cell type, the mode of death, and the spatiotemporal context of the disease. As a fundamental mechanism for maintaining cellular homeostasis, podocytes and renal tubular epithelial cells rely on basal autophagy to selectively clear damaged organelles and misfolded proteins, thereby preserving normal cellular function (80). However, in DKD, glycotoxicity and lipotoxicity often inhibit this beneficial process, resulting in the aberrant accumulation of damaged cellular components and the progressive deterioration of cellular function, ultimately manifesting as progressive proteinuria and deteriorating renal function. Therefore, restoring or enhancing autophagy flux is considered a promising therapeutic strategy (81). In contrast, pyroptosis is an inflammatory form of programmed cell death that involves gasdermin D (GSDMD)-and gasdermin E (GSDME)-mediated plasma membrane pore formation, resulting in cellular content leakage and the robust release of pro-inflammatory mediators (82). In DKD, pyroptosis has been documented in both renal tubular epithelial cells and glomerular resident cells. This process not only directly contributes to renal cell loss but also further amplifies the local inflammatory response through the release of pro-inflammatory mediators, thereby establishing a self-perpetuating cycle between local inflammation and cellular injury (83). Ferroptosis, on the other hand, is an iron-dependent (84, 85), lipid peroxidation-driven form of regulated cell death, and has been demonstrated to contribute to renal tubular injury and renal fibrosis (86). Ferroptosis is mechanistically associated with decreased glutathione peroxidase 4 (GPX4) activity and the accumulation of lipid peroxides; iron-mediated renal tubular injury may be attenuated through modulation of antioxidant defense pathways, including activation of the Nrf2 signaling pathway or GPX4 supplementation (87). Furthermore, apoptosis is particularly prevalent in the early stages of DKD, predominantly affecting podocytes and glomerular endothelial cells (88). Podocyte apoptosis results in podocyte depletion and disruption of foot process architecture, directly impairing glomerular filtration barrier integrity and precipitating proteinuria (89); endothelial cell apoptosis, in turn, disrupts vascular integrity, further exacerbating filtration barrier dysfunction (90); Adequate autophagy flux suppresses apoptosis, whereas impaired autophagy facilitates it (91); caspase-3-mediated cleavage of GSDME can switch apoptosis to pyroptosis (92); selective ferritinophagy degrades ferritin to liberate free iron, thereby providing a substrate for ferroptosis (93). Furthermore, pro-inflammatory mediators released during pyroptosis exacerbate lipid peroxidation and promote ferroptosis (94); ferroptotic byproducts activate the NLRP3 inflammasome, thereby driving pyroptosis (64). Collectively, the distinct forms of programmed cell death in DKD operate in a relatively autonomous yet mechanistically interconnected manner, thereby constituting a complex, integrated network of renal cellular injury.

Hyperglycemia, hemodynamic alterations, oxidative stress, and chronic inflammation cooperatively drive renal injury in DKD. Persistent metabolic stress induces mitochondrial dysfunction, regulated cell death, extracellular matrix accumulation, and progressive renal fibrosis.

### 3.3 Mechanism of action of POSTN in diabetic kidney disease

POSTN has been implicated in DKD through several non-mutually exclusive mechanisms: as a downstream effector of TGF-β and NF-κB signaling, an upstream amplifier of pro-inflammatory and pro-fibrotic circuits, an independent matricellular regulator of ECM assembly, and a marker of fibroblast and myofibroblast activation. These roles are context-dependent, varying with cell type, disease stage, and microenvironment. The following sections distinguish DKD-specific evidence from proposed or context-dependent mechanisms. Mechanistic evidence is presented according to its source: relationships demonstrated in renal cells or tissue are described as established in that setting, whereas pathway models derived from other systems or from expression data alone are identified as proposed or context-dependent.

#### 3.3.1 Expression characteristics and localization of POSTN in diabetic kidney disease

In normal kidneys, renal tissue POSTN protein is present at extremely low baseline levels, primarily in renal tubular epithelial cells and glomerular mesangial cells. In DKD, POSTN is produced by injured tubular epithelial cells, activated fibroblasts and myofibroblasts, mesangial cells, and infiltrating inflammatory cells; the relative contribution of each source varies with disease stage and remains incompletely resolved. In DKD-specific models and human DKD samples, POSTN is upregulated predominantly in the tubulointerstitium, mesangial areas, and fibrotic regions, and its expression correlates with renal functional decline. Related findings in non-diabetic kidney injury models indicate a general association between POSTN and renal fibrosis (95). And do not establish DKD specificity. In diabetic animal models, elevated POSTN expression accompanies progressive renal functional deterioration, histological injury, and interstitial fibrosis. Evidence from other kidney disease settings, including kidney transplantation, extends this association beyond DKD (96). In human DKD specimens, urinary POSTN concentrations increase with DKD severity, and POSTN-deficient mice show attenuated interstitial fibrosis and glomerulosclerosis (97). Furthermore, experimental studies have confirmed that POSTN expression is significantly correlated with pathological renal injury in DM models, with predominant localization within matrix-rich, injured renal regions; targeted interventions to suppress POSTN expression significantly attenuate the expression of fibrosis-associated markers (98).

Localization of POSTN to myofibroblast-rich fibrotic areas suggests that it may also serve as a marker of fibroblast activation. Whether POSTN is merely a marker or a causal driver of DKD progression cannot be determined from expression data alone.

#### 3.3.2 Interaction between POSTN and the NF-κB pathway

A close interaction exists between POSTN and the NF-κB pathway, thereby forming a pro-inflammatory and pro-fibrotic positive feedback network. On the one hand, activation of the NF-κB pathway upregulates POSTN expression; on the other hand, POSTN can reciprocally regulate NF-κB downstream genes, suggesting that it not only participates in the amplification of inflammation but may also serve as a novel biomarker or intervention target (16). Transcriptomic studies have shown that NF-κB activation occurs concurrently with POSTN upregulation and have identified multiple inflammation- and fibrosis-related genes regulated by the NF-κB–POSTN axis, indicating a distinct NF-κB–POSTN regulatory axis in the progression of DKD (16). POSTN gene knockout or knockdown reduces the expression of pro-inflammatory markers and alleviates histological fibrosis, supporting a critical role for POSTN in maintaining the inflammatory-fibrotic response (17). However, direct evidence that POSTN acts as an upstream amplifier of NF-κB in human DKD is limited, and the relative contribution of each direction remains unresolved.

#### 3.3.3 The role of POSTN in the TGF-β signaling pathway

In DKD-relevant high-glucose and TGF-β1-stimulated renal tubular cells, POSTN is induced and reciprocally amplifies TGF-β-mediated pro-fibrotic responses. Findings from non-renal fibrotic models, such as adventitial remodeling, provide additional biological context and should not be regarded as direct DKD evidence (99). In renal tubular epithelial cells stimulated by TGF-β1 in combination with high glucose, increased POSTN expression is accompanied by changes in typical EMT markers, manifested as upregulation of α-smooth muscle actin (α-SMA) and downregulation of E-cadherin. In contrast, knocking down POSTN or neutralizing it with antibodies partially reverses these changes, suggesting that POSTN may act as a downstream effector of TGF-β signaling in this context. High glucose induces TGF-β, which upregulates POSTN; POSTN may then contribute to EMT-like changes and ECM synthesis via the integrin–p38/MAPK axis. In diabetic POSTN-deficient mice, interstitial fibrosis and glomerulosclerosis are attenuated, together with improved expression of EMT-related markers (97). Furthermore, *in vitro* experiments with human kidney-2 (HK-2) cells demonstrated that exogenous POSTN synergistically enhances ECM deposition under TGF-β stimulation, highlighting POSTN’s pivotal role as a downstream effector molecule of TGF-β in driving renal fibrosis. These findings support a contributory role for POSTN in tubular EMT-like changes, but they do not establish that tubular epithelial cells directly generate interstitial myofibroblasts through classical EMT.

#### 3.3.4 The p38/MAPK pathway mediates POSTN-induced EMT-like changes

The p38-MAPK pathway is a key intracellular signaling pathway through which EMT-like changes. Under high-glucose conditions, POSTN induces EMT-like phenotypic changes by activating the p38-MAPK signaling pathway. *In vitro* studies have shown that in high-glucose-stimulated human HK-2 renal tubular epithelial cells, elevated POSTN expression correlates with increased p38 phosphorylation levels, accompanied by upregulation of α-SMA and downregulation of E-cadherin (98). Treatment with specific p38 inhibitors significantly reduces POSTN and α-SMA expression and restores E-cadherin levels, suggesting that p38/MAPK activation contributes to POSTN-induced EMT-like changes. Further mechanistic studies revealed that p38 inhibitors not only reverse the EMT-like phenotype induced by POSTN overexpression but also reduce the synthesis and secretion of ECM components under high-glucose conditions. Notably, under the combined stimulation of TGF-β1 and high glucose, blocking p38/MAPK signaling similarly attenuates the pro-fibrotic effects downstream of POSTN, suggesting that p38/MAPK may serve as a potential signaling node in POSTN-related tubular responses and represents a potential therapeutic target for intervention in renal fibrosis in DKD (100). p38/MAPK activation appears to be a functional step in POSTN-induced EMT-like changes *in vitro*; *in vivo* causality and human relevance require further validation.

#### 3.3.5 The role of POSTN in extracellular matrix remodeling

As a matrix protein, POSTN interacts directly with ECM components such as type I collagen through its FAS1 domain, promoting collagen cross-linking and assembly, and renal tissue POSTN protein levels are closely associated with excessive ECM deposition in DKD. In general matrix biology and non-renal models, POSTN binds to integrin receptors, thereby mediating cell adhesion and migration, and promoting the differentiation of fibroblasts into myofibroblasts (101, 102); Simultaneously, POSTN activates the FAK and PI3K/Akt pathways, collaborates with tenascin-C to remodel the ECM, and interacts functionally with growth factors such as fibroblast growth factor (FGF) and vascular endothelial growth factor (VEGF). In these non-renal systems, POSTN also regulates the activity of matrix metalloproteinases (MMP)-2/MMP-9 and thereby ECM degradation (103). In DKD-specific models, POSTN stabilizes ECM structure during renal injury, and POSTN intervention reduces the accumulation of fibrotic ECM (97). Bioinformatics analysis identifies POSTN as a hub gene consistently upregulated in both glomerular and tubular DKD datasets, supporting its involvement in the ECM remodeling network in DKD (104).

In summary, POSTN has been proposed to form an inflammation-fibrosis coupling network in DKD through multiple signaling pathways, including NF-κB, TGF-β, and p38/MAPK (**Figure 3**).

**Figure 3:**
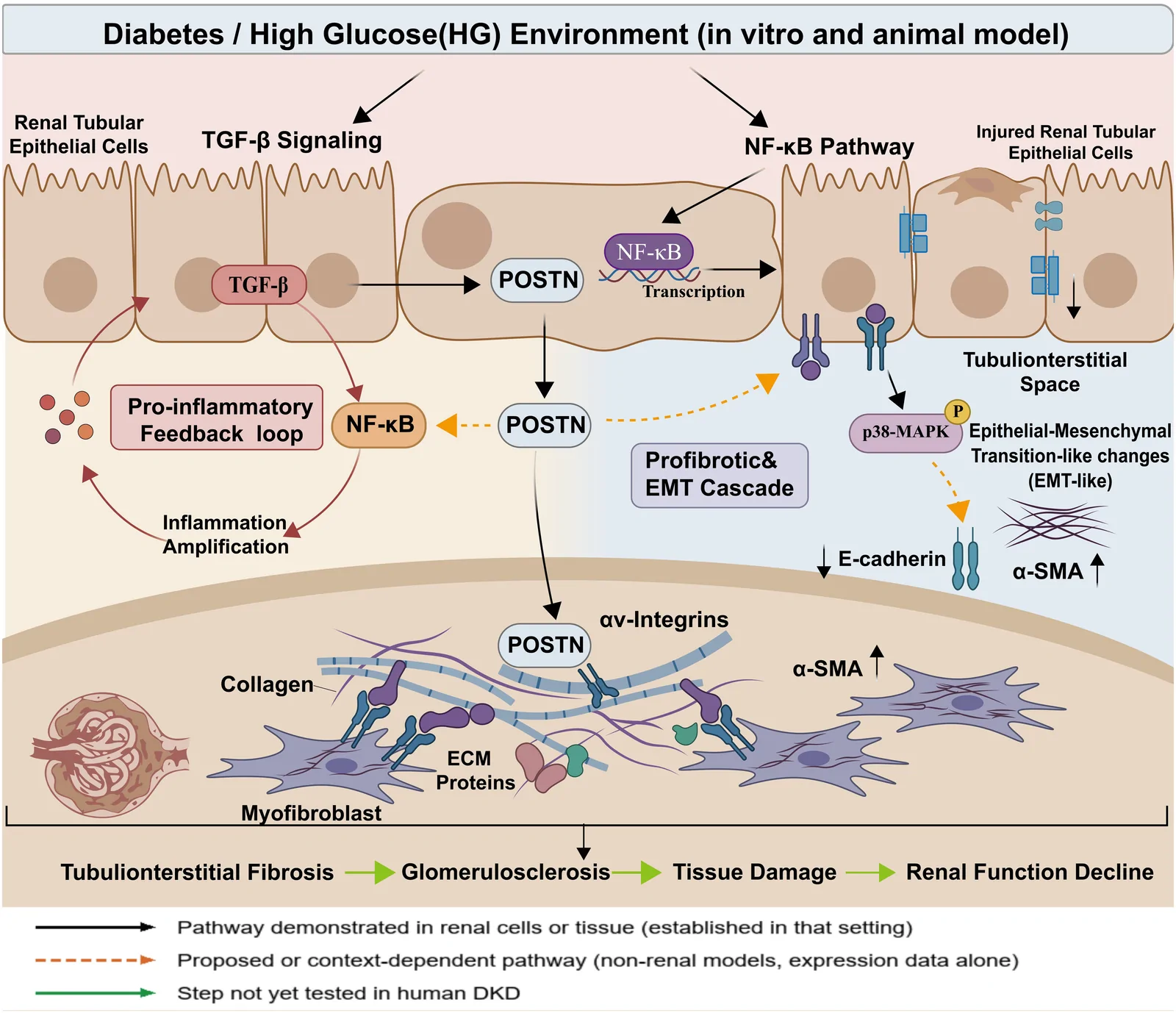
Proposed POSTN-centered inflammatory and fibrotic signaling in DKD.

In renal tubular cells and in diabetic animal models, hyperglycemia and inflammatory stimuli induce POSTN expression. Evidence from renal cells supports POSTN-dependent activation of integrin/FAK and p38/MAPK signaling and EMT-like changes *in vitro*, and POSTN inhibition reduces fibrosis in animal models. Whether POSTN amplifies NF-κB signaling, and how far these pathways drive progressive renal fibrosis in patients, remains proposed or context-dependent because direct human DKD evidence is limited.

## 4 Population-based evidence on POSTN as a candidate biomarker for diabetic kidney disease

### 4.1 Key findings from cross-sectional and case-control studies

Current population-based evidence regarding the association between POSTN and DKD primarily comes from cross-sectional and case-control studies. Varatharajan et al. included 160 participants and systematically evaluated the diagnostic performance of urinary POSTN and other kidney injury markers. The results showed that urinary POSTN levels increased in a stepwise manner, were positively correlated with the urine albumin-to-creatinine ratio (ACR), and negatively correlated with eGFR (105). Another cross-sectional study involving 137 patients with type 2 diabetes mellitus (T2DM) evaluated the distribution of urinary POSTN across different albuminuria stages. The study found that urinary POSTN levels were significantly elevated compared with healthy controls even during the normoalbuminuria phase and continued to rise as albuminuria severity increased. Urinary POSTN was positively correlated with ACR and the tubular injury marker neutrophil gelatinase-associated lipocalin (NGAL), suggesting that urinary POSTN is a candidate early marker of tubular damage that requires prospective validation (106). Taha et al. employed a case-control design involving 102 T2DM patients and found that urinary POSTN levels were significantly higher in the T2DM group than in the control group, and significantly higher in the poorly controlled group than in the well-controlled group. Furthermore, urinary POSTN was positively correlated with ACR and negatively correlated with eGFR (107). Urinary POSTN rose progressively from normoalbuminuria to microalbuminuria and then to macroalbuminuria across these cross-sectional studies; whether this gradient predicts progression has not been tested prospectively. Additionally, urinary POSTN showed a significant positive correlation with ACR and a weak to moderate negative correlation with eGFR. In that cohort, urinary POSTN remained associated with DKD after adjustment for conventional variables, and confirmation in prospective studies is required. As a secreted protein, POSTN is upregulated and released into body fluids upon renal injury, making it a potential biomarker that can be monitored via non-invasive methods such as urine testing (108). These findings suggest that POSTN may be used for early identification of high-risk DKD populations.

Across the available human studies, three consistent observations emerge. First, urinary POSTN is elevated in patients with DKD and correlates positively with albuminuria and negatively with eGFR. Second, the association is graded across normoalbuminuria, microalbuminuria, and macroalbuminuria. Third, urinary POSTN is often higher in patients with poor glycemic control. These studies are predominantly cross-sectional, single-center, and conducted in patients with type 2 diabetes, and few report independent validation cohorts, adjustment for urine albumin-to-creatinine ratio (UACR) and eGFR, area under the curve (AUC), specificity, positive and negative predictive value, calibration, or clinically relevant cutoffs. The current evidence supports an association between POSTN and DKD severity; diagnostic or prognostic superiority over conventional markers remains unestablished.

### 4.2 Population distribution characteristics

Preliminary reports have described the distribution of urinary POSTN levels across different subgroups. First, glycemic control status significantly influences urinary POSTN levels. A cross-sectional study by Taha et al. involving 102 patients with type 2 diabetes showed that, after stratification by glycated hemoglobin (HbA1c), urinary POSTN levels were significantly higher in the group with poor glycemic control (HbA1c >6.5%) compared to the group with good control (HbA1c ≤6.5%), suggesting that glycemic control is a key factor influencing urinary POSTN levels (107). The type of diabetes is also associated with POSTN expression; existing studies have primarily focused on patients with T2DM, while epidemiological data on POSTN distribution in patients with type 1 diabetes mellitus (T1DM) remain relatively limited (109). Urinary POSTN levels show an upward trend with disease progression. Patel et al. reported from a tertiary hospital that urinary POSTN levels in DKD patients increase with chronic kidney disease (CKD) stage progression, and that urinary POSTN levels in ESRD patients on hemodialysis are significantly higher than those in patients with early-stage DKD (109). Duan et al. also found in a DKD model that renal tissue POSTN protein levels were detectable and elevated in the early stages of the disease; however, data on its distribution across disease stages in the general population require further validation (65). Notably, racial and regional differences should not be overlooked. Existing studies primarily originate from Asian regions such as China, India, and Iraq, as well as some European and American regions, but lack systematic cross-racial and cross-regional comparisons (110). Baseline serum POSTN levels and disease-related change patterns across different ethnic groups remain unclear. Furthermore, age and sex may also modulate serum POSTN levels; however, existing cross-sectional studies have not systematically reported the independent effect of age on serum POSTN levels. Although some studies have included age as a covariate in multivariate analyses, the age effect is rarely reported in isolation (111). Evidence regarding sex differences is similarly insufficient; most study samples had an imbalanced male-to-female ratio and did not conduct sex-stratified analyses. Given the known sex differences in the incidence and progression rate of DKD, sex differences in serum POSTN levels warrant further investigation. The main studies evaluating POSTN expression and its clinical associations in DKD are summarized in **Table 1**.

### 4.3 Analysis of clinical factors affecting serum and urinary POSTN levels

Identifying clinical factors influencing serum and urinary POSTN levels is crucial for understanding its epidemiological significance and clinical utility. Glycemic control is a major factor affecting urinary POSTN levels. Existing evidence indicates that poor glycemic control is independently associated with elevated urinary POSTN, and multivariate analyses confirm that glycemic control status is an independent influencing factor, supporting the role of POSTN in glucose metabolism-mediated renal damage (107). However, current population-based evidence regarding the dose-response relationship between continuous HbA1c levels and POSTN, as well as the association between glycemic variability and POSTN, remains insufficient. Additionally, while POSTN is expressed in the cardiovascular system and is associated with myocardial fibrosis, the independent impact of cardiovascular complications on serum POSTN levels in the DKD population has not been systematically evaluated (116). Furthermore, pharmacotherapy is another important influencing factor. As standard treatments for DKD, ACEIs and ARBs may theoretically reduce renal tissue POSTN protein levels by alleviating renal fibrosis; however, existing studies have not systematically evaluated the impact of these medications on renal tissue POSTN protein levels (117). The renoprotective effects of different antidiabetic drugs vary; whether these drugs influence renal tissue POSTN protein levels and whether changes in these levels mediate their renoprotective effects warrant further investigation. From a histopathological perspective, both animal experiments and clinical studies have demonstrated a close association between POSTN and renal interstitial fibrosis. Clinical cross-sectional studies have also observed that urinary POSTN levels rise with renal injury progression (105). POSTN is also expressed in bone, lung, and adipose tissue, so circulating levels may be influenced by obesity, cardiovascular disease, bone and pulmonary disease, systemic fibrosis, and reduced renal clearance; these determinants have not been systematically adjusted for in DKD cohorts.

## 5 Application of POSTN in the diagnosis of diabetic kidney disease

### 5.1 Comparison of POSTN with traditional diagnostic markers for diabetic kidney disease and its value in early diagnosis

Traditional diagnosis of DKD primarily relies on indicators such as albuminuria, serum creatinine, and eGFR; however, these parameters typically become abnormal only after renal damage has progressed to a certain stage, thereby missing the optimal window for early intervention (5). POSTN has been proposed as a candidate biomarker that may complement conventional indicators, but the supporting human evidence is largely cross-sectional and single-center, and prospective validation is still required.

The ability to distinguish proteinuria stages and to detect DKD at an early stage is central to evaluating the clinical value of POSTN. As urinary albumin excretion increases progressively, urinary POSTN exhibits a parallel upward trend and is positively correlated with ACR (106). Importantly, elevated urinary POSTN levels have been observed in some patients with DM during the normoalbuminuria phase, suggesting that urinary POSTN may rise before the onset of albuminuria in some patients, a pattern that requires prospective confirmation (109). This characteristic stems from differences in pathophysiological mechanisms: albuminuria reflects disruption of the glomerular filtration barrier; whereas POSTN, as a matricellular protein, may additionally reflect tubulointerstitial fibrosis and EMT-like tubular responses. POSTN may therefore provide pathological information complementary to albuminuria and could help assess damage extent and fibrosis risk in DKD. Biological mechanisms provide a rationale for evaluating POSTN in early-stage disease. Under high glucose and TGF-β1 stimulation, POSTN is rapidly upregulated in renal tubular epithelial cells; similarly, in the HK-2 cell model, high glucose combined with TGF-β1 induces POSTN expression. This process accompanies the onset of renal fibrosis and precedes detectable histological fibrosis and renal failure in experimental models (98). POSTN also acts as a pathogenic factor: it promotes ECM deposition, EMT-like changes, and inflammatory amplification via integrin receptors, forming a pro-fibrotic feedback loop (16). Animal studies have further demonstrated that blocking POSTN reduces albuminuria, glomerulosclerosis, and interstitial fibrosis (97).

POSTN may rise earlier than serum creatinine and eGFR in some patients. Urinary POSTN levels are negatively correlated with eGFR and positively correlated with serum creatinine, and are significantly elevated in patients with declining renal function. One cross-sectional study reported lower urinary POSTN in CKD stage 1 than in stages 3–4, suggesting that urinary POSTN may track renal impairment severity; this requires prospective confirmation (112). Creatinine-based eGFR is relatively insensitive to early nephron loss and may remain within the reference range despite substantial structural injury, and early DKD can be missed when eGFR is used alone (118); In contrast, urinary POSTN levels rise early in the course of structural renal damage through fibrosis processes mediated by signaling pathways such as TGF-β/p38/MAPK and NF-κB, which may allow earlier detection; this remains to be demonstrated prospectively. In the available cross-sectional studies, the reported sensitivity of urinary POSTN was comparable to that of ACR (105). In that hospital-based study, the reported sensitivities of urinary POSTN, ACR, NGAL, and kidney injury molecule-1 (KIM-1) were 80%, 80%, 75.8%, and 63.3%, respectively (105). In one cross-sectional tertiary-center study, urinary POSTN was associated with renal injury; whether it provides incremental diagnostic value beyond ACR and eGFR has not been established (109). Current research is limited by the lack of large-scale prospective cohort validation and standardized cutoff values. Most existing literature consists of small-sample cross-sectional studies, and no unified POSTN reference range or diagnostic threshold has been established, as cutoff values reported in different studies vary. Moreover, key diagnostic parameters such as the specificity, positive predictive value, and negative predictive value of POSTN testing have not been fully reported, which limits its clinical application.

Available studies suggest that urinary POSTN may have diagnostic value in DKD, although the evidence base is heterogeneous. Reported sensitivity ranges from approximately 80% to 100%, with one study reporting an optimal cutoff of 37.5 ng/mL and 80% sensitivity (110). Specificity, AUC, positive and negative predictive value, calibration, and external validation are inconsistently reported. Most studies do not adjust for UACR and eGFR, and none has demonstrated that POSTN adds diagnostic value beyond these conventional markers. Urinary POSTN currently qualifies as a candidate diagnostic biomarker that requires prospective validation.

### 5.2 Research progress in POSTN detection technologies

Advances in POSTN detection technology are relevant to its evaluation as a candidate biomarker for early diagnosis and monitoring in DKD. In recent years, various detection methods have been widely applied in clinical and basic research, each with distinct characteristics. Among these, the enzyme-linked immunosorbent assay (ELISA) is currently the most commonly used method for quantitative POSTN detection in clinical research (119). This technique utilizes specific antibodies to recognize POSTN in urine or serum, is simple to perform, highly sensitive, and suitable for large-scale sample testing. In one study, receiver operating characteristic (ROC) analysis of urinary POSTN measured by ELISA yielded 80% sensitivity at a cutoff of 37.5 ng/mL for DKD; specificity and AUC were not reported. Furthermore, the non-invasive nature of urine samples makes repeated measurement feasible in research settings, as urinary POSTN levels rise with increasing DKD severity and are positively correlated with ACR (105). Unlike ELISA, immunohistochemistry and immunostaining techniques play an irreplaceable role in POSTN detection at the tissue level, providing visual representation of POSTN’s spatial distribution within renal tissue, particularly its expression patterns in the expanded mesangial region, tubulointerstitial space, and fibrotic areas (120). Combined with fibrosis markers such as Sirius red staining, these techniques allow for more accurate evaluation of the correlation between POSTN expression and renal fibrosis, providing crucial evidence for pathological assessment (120). Furthermore, at the cellular and molecular levels, Western blotting and immunofluorescence techniques are widely applied. The former can accurately quantify POSTN protein levels in tissue or cell lysates and distinguish between different protein subtypes, making it particularly suitable for signaling pathway research and evaluating drug intervention effects, whereas the latter allows for real-time observation of POSTN expression and localization at the cellular level and is often used in conjunction with other protein markers to study pathological processes such as EMT. Meanwhile, reverse-transcription polymerase chain reaction (RT-PCR) technology offers unique advantages in detecting POSTN at the mRNA level, reflecting dynamic changes in gene transcriptional regulation. This technique is frequently combined with protein detection methods to comprehensively evaluate POSTN expression regulation at both the transcriptional and translational levels, and its high sensitivity enables the detection of early gene expression changes, providing a crucial tool for studying POSTN dynamics during DKD progression (121). Finally, novel aptamer technology represents an innovative direction in POSTN detection and intervention. POSTN-binding DNA aptamers exhibit higher binding affinity and lower immunogenicity than traditional antibodies (122). Although currently used primarily in therapeutic intervention studies, their highly specific binding properties offer potential for developing novel detection methods. Furthermore, aptamer technology has been demonstrated to effectively inhibit POSTN-mediated renal fibrosis in DM mouse models, indicating promising application prospects.

### 5.3 Comparison of diagnostic performance between serum and urine POSTN

#### 5.3.1 Serum POSTN

As a potential circulating biomarker, serum POSTN theoretically reflects systemic fibrotic burden. Its origin is complex; aside from damaged kidneys, organs such as the heart, lungs, and bones also secrete POSTN into the bloodstream under pathological conditions. Consequently, serum POSTN levels are influenced by the combined effects of multi-organ status and systemic metabolic clearance, resulting in a relatively indirect association with specific renal lesions. Nevertheless, several studies have explored the diagnostic value of serum POSTN in DKD. Research in kidney transplant recipients, a non-DKD population, found a median serum POSTN level of 959 ng/mL compared with 1176 ng/mL in healthy volunteers (115). This observation suggests that serum POSTN levels in this non-DKD population may fall in response to immunosuppressants or impaired renal function, and it warrants further investigation (115). Serum POSTN has been reported as a fibrogenic marker, and in one series it improved the non-invasive prediction of renal fibrosis severity when combined with the renal resistance index and shear-wave elastography in patients with DKD (113). A clinical study conducted among male T2DM patients in Iraq found that serum POSTN levels were significantly higher in DKD patients than in T2DM controls without kidney disease, suggesting that it warrants evaluation as an early screening marker for DKD (114). Furthermore, a cross-sectional study found that dynamic changes in serum POSTN were positively correlated with declining creatinine clearance, suggesting that it may reflect progressive renal function loss (113). However, studies on serum POSTN face numerous limitations. There is significant heterogeneity in study populations, inconsistencies in detection methods and reporting units, and most studies employ cross-sectional designs, lacking direct evidence from histological comparisons with renal biopsy specimens. Therefore, the clinical utility of serum POSTN as a specific biomarker for DKD requires validation through large-scale, prospective, DKD-specific studies.

#### 5.3.2 Urinary POSTN

POSTN is not kidney-specific. Serum POSTN reflects multi-organ sources, including heart, lung, bone, and tumor stroma, and is affected by renal clearance. Urinary POSTN may be enriched for renal production but is not exclusively kidney-derived; filtered systemic POSTN, tubular handling, urine concentration, and assay variability all influence measured concentrations, so urinary POSTN should be reported with urinary creatinine normalization. Urinary POSTN is likely derived at least in part from injured tubular epithelial cells and interstitial myofibroblasts, and urinary POSTN may more closely reflect local renal production than serum POSTN, but this has not been validated (123). Furthermore, urine collection is non-invasive and highly reproducible, making it a suitable candidate biomarker for early screening and disease monitoring in DKD (118). Several clinical studies report that urinary POSTN is elevated in DKD. One study used ROC curve analysis to define a cutoff of 37.5 ng/mL for urinary POSTN in diagnosing DKD, with a sensitivity of 80%, comparable to ACR and superior to KIM-1; specificity, AUC, positive and negative predictive value, assay standardization, and external validation remain unavailable (105, 109). Elevated urinary POSTN levels in patients with T2DM correlate with the renal tubular damage marker NGAL, which is consistent with a possible role as a non-invasive tubular injury marker (124). Findings from animal models and clinical samples indicate that urinary POSTN concentrations increase with DKD severity and are positively correlated with the degree of renal interstitial fibrosis (97). Related studies further report that urinary POSTN levels are elevated in the normoalbuminuria phase compared with healthy controls and rise with albuminuria severity (109). These findings suggest that urinary POSTN may reflect DKD severity and could be useful for detecting early tubulointerstitial damage before the onset of albuminuria, pending prospective validation.

#### 5.3.3 Comparison of serum and urinary POSTN

In summary, urinary POSTN shows a consistent upward trend and a close correlation with kidney injury severity across multiple studies, whereas serum POSTN data are scarcer and more inconsistent. Serum POSTN is influenced by extrarenal production and by reduced renal clearance as GFR falls, so it reflects renal pathology only indirectly. Urinary POSTN is enriched for locally produced protein from injured tubules and interstitium, while spot urine concentrations also depend on hydration and urine concentration, which makes creatinine normalization necessary. Assay platform, sample handling, and intra-day biological variation add further variability, and longitudinal data remain scarce. Urinary POSTN has the stronger DKD-specific evidence base of the two; both require standardized assays and prospective validation before clinical use.

## 6 Research progress on POSTN in the treatment of diabetic kidney disease

### 6.1 Preclinical studies on POSTN-targeted therapeutic strategies

As understanding of the critical role of POSTN in DKD pathogenesis deepens, POSTN-targeted strategies have become an active area of preclinical investigation. All evidence summarized below is derived from animal or *in vitro* studies unless otherwise specified. Several approaches have shown anti-fibrotic effects in diabetic animal models, defining candidate directions for DKD treatment. Among these, POSTN-binding aptamer technology provides a direct route to POSTN inhibition. This aptamer is a high-affinity nucleic acid ligand that specifically recognizes and neutralizes extracellular POSTN, thereby blocking its interaction with integrin receptors. In diabetic mice, aptamer treatment reduces blood urea nitrogen levels and alleviates tubulointerstitial fibrosis and the expression of fibronectin and type I collagen (125). Similarly, an anti-POSTN antibody attenuated renal fibrosis in mice with unilateral ureteral obstruction (UUO), reducing fibrosis- and inflammation-related gene expression (126). These animal and cell-culture findings suggest that POSTN may also act outside the kidney; whether antibody therapy improves metabolic control and renal outcomes in patients remains untested. Genetic intervention techniques, such as knockdown or silencing of POSTN expression, provide powerful tools for exploring the causal role of POSTN. In a DKD model using POSTN knockout mice, after 20 weeks of observation, the knockout group exhibited better renal protection compared with the wild-type control group, with reduced urinary albumin excretion, attenuated glomerulosclerosis and interstitial fibrosis, and decreased expression of fibrosis markers. Furthermore, POSTN knockdown improved glucose regulation in DM mice and preserved islet integrity and insulin expression, indicating that POSTN plays a key role in the metabolic-renal axis in DKD (97). Additionally, siRNA-mediated POSTN knockdown alleviates pathological changes and fibrosis in animal models of diabetic renal injury (17); this approach has not been tested in humans. In high-glucose-stimulated human renal tubular cells *in vitro*, inhibition of p38/MAPK attenuates EMT-like changes, at least in part by indirectly downregulating POSTN expression (100). In summary, animal and *in vitro* evidence suggests that targeting POSTN and its associated pathways may delay DKD progression; no human trial has yet tested this.

Preclinical intervention studies can be grouped into four strategies: aptamer-mediated neutralization, anti-POSTN antibodies, genetic knockdown or knockout, and indirect pathway inhibitors. Across these studies, POSTN inhibition is consistently associated with reduced albuminuria, attenuated glomerulosclerosis and interstitial fibrosis, and decreased expression of fibrosis markers in diabetic animal models. These findings are limited by species differences, short follow-up, single-model designs, and the absence of human validation. POSTN remains a preclinical candidate target that has not been validated clinically. Preclinical studies investigating therapeutic targeting of POSTN are summarized in **Table 2**.

### 6.2 Combination therapy of POSTN with existing treatments for diabetic kidney disease

Current treatment for DKD has evolved into a model centered on multi-target combination therapy, including RAAS inhibitors, SGLT2 inhibitors, GLP-1 receptor agonists, and non-steroidal selective mineralocorticoid receptor antagonists; the clinical benefit of these agents is established in patients with DKD. In contrast, all evidence for combining them with POSTN-targeted therapy comes from animal and *in vitro* studies. Combining POSTN-targeted therapy with these existing treatments is hypothesized to produce complementary effects on the basis of preclinical rationale, but clinical synergy has not been demonstrated (128). The proposed combination with RAAS inhibitors rests on preclinical mechanistic reasoning: in animal and cell models, RAAS inhibition reduces TGF-β signaling and may thereby lower POSTN expression, and residual POSTN activity is proposed to sustain fibrosis (129). In diabetic mice, POSTN-binding aptamers reduce tubulointerstitial fibrosis (125); no study has tested this combination in patients. In patients with DKD, SGLT2 inhibitors and GLP-1 receptor agonists provide metabolic and renal protection (130). Whether these agents lower POSTN levels in humans has not been tested directly. In animal and cell models, improved glycemia is accompanied by lower POSTN expression and POSTN knockdown preserves β-cell function (Section 6.1); on this basis a positive feedback between glycemic control and POSTN activity has been proposed, but it remains a hypothesis. Observational human data show that urinary POSTN levels are higher in patients with poor glycemic control (107); this association does not establish that POSTN-targeted therapy improves glycemic control. Furthermore, POSTN is a candidate therapeutic response biomarker that requires prospective validation. Patients with high baseline urinary POSTN levels might derive additional benefit from targeted therapy, and a decrease in POSTN levels following treatment could potentially indicate anti-fibrotic efficacy. In summary, every statement above about combining POSTN-targeted therapy with current treatments rests on animal and *in vitro* evidence; the efficacy and safety of any such combination in humans have not been evaluated in clinical trials.

## 7 Controversies and prospects regarding POSTN in the study of diabetic kidney disease

### 7.1 Core controversies regarding the mechanism of action of POSTN

Several controversies remain regarding the exact mechanism of action of POSTN in DKD. First, debate exists over whether POSTN acts through “direct” or “indirect” pathways. Some evidence suggests that POSTN directly drives inflammation and fibrosis via renal-specific pathways such as NF-κB (16); conversely, other studies found that POSTN deficiency simultaneously improves pancreatic β-cell function and glucose regulation, suggesting that its renoprotective effects may partially stem from improved metabolic control (97). This discrepancy may arise from differences in animal models and intervention timing; studies involving tubule-specific POSTN knockout that avoid interfering with systemic metabolism are lacking. Second, controversy exists regarding whether POSTN acts as a “driver” or “responder” in fibrosis. Most studies suggest that POSTN is a downstream effector of TGF-β; however, evidence also indicates that POSTN can activate TGF-β expression through feedback, forming a positive feedback loop (131). Furthermore, POSTN may exert opposing effects in different pathological contexts. In non-DKD cystic kidney disease, MMP-9-mediated POSTN cleavage appears protective; whether this applies to DKD is unknown (127). Finally, although several clinical studies report higher POSTN levels in DKD, sensitivity and specificity vary across populations and diabetes types, and standardized assays and uniform reference ranges are lacking. These controversies suggest that the role of POSTN in DKD exhibits complex context-dependence, and that more refined *in vivo* models and standardized population-based studies are needed to resolve these contradictions.

POSTN research faces numerous methodological and ethical challenges. Regarding detection techniques, variations in ELISA kits, sample processing protocols, and normalization methods across studies hinder direct result comparison (132); serum POSTN levels exhibit significant intra-day fluctuations, and non-standardized sampling times may introduce bias (133). Fully validated international standard reference materials and unified quality control systems are lacking. Regarding animal models, although gene-knockout mice and chemically induced DKD models provide important insights, they cannot fully replicate human disease complexity (134). POSTN function in the microenvironment exhibits substrate specificity, and findings from simple models often cannot be directly extrapolated to clinical settings. Human studies also require standard safeguards for informed consent, biobanking, data sharing, and genetic privacy. Furthermore, most mechanistic studies focus on single signaling pathways, whereas POSTN, as a multifunctional matrix protein, participates in multiple intersecting pathways; simplified models cannot fully reflect its complex *in vivo* functions. Insufficient reproducibility across studies, small sample sizes, and lack of independent validation cohorts limit the field’s development. Establishing pre-registered study protocols, making raw data publicly available, and advancing multicenter prospective validation are essential for improving research quality.

Collectively, these controversies indicate that POSTN is best conceptualized as a context-dependent matricellular node that can act as both driver and responder in DKD.

### 7.2 Safety and translational bottlenecks of POSTN-targeted therapy

Although preclinical studies consistently confirm that POSTN inhibition reduces renal fibrosis, translating this into clinical applications faces safety concerns. POSTN participates in tissue development, injury repair, and wound healing under physiological conditions; while POSTN protein levels are low in healthy adult tissues, they are rapidly upregulated in conditions such as myocardial infarction and skin trauma to promote repair. Bone metabolism provides a further non-renal example of these physiological roles (135). Systemic, long-term POSTN inhibition may interfere with these protective processes, leading to adverse consequences. As previously mentioned, POSTN’s role varies significantly across different pathological contexts; in non-DKD cystic kidney disease, its moderate presence appears protective (127). This suggests that simple systemic neutralization strategies may carry risks, necessitating tissue-specific delivery or selective intervention. Furthermore, all current evidence for POSTN-targeted therapies comes from preclinical studies; no drugs have entered human clinical trials. Translating findings from animal models to humans presents significant challenges. Animal DKD models typically have short disease durations and single pathologies, hindering simulation of metabolic memory effects, multiple complications, and individual variations associated with long-term diabetes in humans. Therefore, future safety assessments must encompass multiple organ systems, pharmacokinetic-pharmacodynamic studies, and wound healing monitoring.

Future POSTN research should leverage cutting-edge technologies to deepen mechanistic understanding and facilitate clinical translation. At the omics level, transcriptomics has identified POSTN as a core hub gene in DKD, but proteomics and metabolomics research remains scarce (104). Integrating multi-omics data will help elucidate POSTN’s post-translational modifications, protein interaction networks, and their impact on metabolic pathways (104). Weighted gene co-expression network analysis and machine learning algorithms can construct clinical prediction models featuring POSTN, enabling personalized assessment of DKD progression risk. Novel platforms such as nanotechnology, microfluidic chips, and electrochemical biosensors enable development of point-of-care devices, enhancing urinary POSTN detection convenience and sensitivity. Simultaneously, establishing standardized sampling procedures and reference ranges is essential for clinical application. Exosome-encapsulated POSTN may offer higher stability and tissue specificity than its free form, and its quantification may provide more precise disease information. Evidence for exosomal POSTN comes from retinal neovascularization and other non-renal settings, and this field remains largely unexplored in DKD (136).

### 7.3 Innovative directions and combination applications for therapeutic strategies

Clinical translation of POSTN-targeted therapy requires multi-level innovation. Anti-POSTN monoclonal antibodies, nanobodies, and high-affinity aptamers have shown promise; small-molecule inhibitors targeting pathways such as POSTN-integrin/FAK, POSTN-NF-κB, and p38/MAPK also represent viable indirect intervention strategies. Gene editing technologies such as clustered regularly interspaced short palindromic repeats (CRISPR) may be explored. Investigating potential complementary effects of POSTN intervention with existing DKD-targeted medications is a rational preclinical strategy. SGLT2 inhibitors may indirectly downregulate POSTN expression by improving the metabolic environment, and GLP-1 receptor agonists may exert anti-inflammatory and anti-fibrotic effects that intersect with the POSTN pathway (137). Future clinical trials should evaluate additional benefits of anti-POSTN interventions in patients with elevated urinary POSTN levels and progressive proteinuria receiving standard therapy. Furthermore, POSTN’s dual nature in tissue repair suggests that spatially and temporally controlled interventions may facilitate transition from pathological fibrosis to functional regeneration. Comprehensive DKD care must also address complications that develop alongside fibrosis, including anemia (11).

POSTN’s biomarker properties suggest several hypothetical applications in personalized DKD treatment. The proposals below outline a future research agenda; none is an established clinical recommendation. Regarding risk stratification, classifying patients into low-, medium-, and high-risk groups on the basis of urinary POSTN levels has been proposed. Whether more intensive monitoring of patients with high POSTN expression improves outcomes or resource allocation has not been tested. For treatment matching, the hypothesis that patients with high POSTN expression may derive greater benefit from anti-inflammatory or anti-fibrotic agents, including biologics directed against POSTN, remains untested. An algorithm linking POSTN expression profiles to treatment regimens is a proposed future tool for moving from empirical toward precision medicine. For treatment monitoring, POSTN has been proposed as a candidate marker of anti-fibrotic treatment response rather than an established monitoring tool. A hypothetical study design would measure urinary POSTN at baseline and repeat the measurement 1–3 months after treatment initiation, on the assumption that this interval might detect a treatment effect earlier than changes in eGFR or proteinuria; whether such a schedule is feasible, reproducible, or clinically informative in DKD is unknown and requires prospective testing. Serial POSTN monitoring has been used to track treatment response in idiopathic pulmonary fibrosis, whereas equivalent DKD data are not yet available (138). Machine learning models incorporating POSTN, clinical parameters, other biomarkers, and genetic risk scores could in future support prediction of DKD progression risk, time to ESRD, and treatment response, and might help identify high-risk populations for enrolment in clinical trials. Although prospects are promising, standardization of POSTN testing, control of intra-day variability, and large-scale prospective validation remain obstacles to clinical implementation, and no POSTN-based monitoring schedule is currently recommended for clinical practice.

## 8 Conclusion

As a multifunctional matricellular protein, POSTN plays a dual role in DKD development and progression. It is a candidate biomarker reflecting tubulointerstitial fibrosis and a potential pathogenic contributor to inflammation-fibrosis coupling. Cross-sectional clinical studies generally report positive correlations between urinary and serum POSTN levels and proteinuria severity, the rate of renal function decline, and renal histopathological damage in DKD patients, but these associations have not been validated prospectively and no assay or cutoff has been standardized. Urinary POSTN is also elevated during the normoalbuminuria phase in several cohorts, which supports its evaluation as a candidate early biomarker for diagnosis and risk stratification. Preclinical studies suggest that targeting POSTN may reduce renal fibrosis; however, clinical efficacy, safety, and target engagement in humans remain unvalidated.

POSTN’s clinical translation still faces unresolved questions about its mechanism of action, the absence of standardized assays and cutoffs, and the unproven safety of long-term inhibition. Priorities for future work are a standardized POSTN assay, large-scale prospective cohorts, tissue-specific delivery, and characterization of POSTN splicing variants in DKD. In summary, POSTN is consistently associated with renal fibrosis and DKD severity in preclinical models and small human studies. However, DKD-specific human evidence remains limited, and findings from IgA nephropathy, kidney transplantation, cystic kidney disease, UUO, 5/6 nephrectomy, and non-renal systems should not be extrapolated to DKD without validation. POSTN therefore remains a promising preclinical candidate biomarker and therapeutic target, not yet clinically validated.
